# Determination of sialic acid in saliva by means of surface-enhanced Raman spectroscopy as a marker in adnexal mass patients: ovarian cancer vs benign cases

**DOI:** 10.1186/s13048-018-0433-9

**Published:** 2018-07-24

**Authors:** José de Jesús Zermeño-Nava, Marco Ulises Martínez-Martínez, Ana Laura Rámirez-de-Ávila, Aida Catalina Hernández-Arteaga, Ma. Guadalupe García-Valdivieso, Alondra Hernández-Cedillo, Miguel José-Yacamán, Hugo Ricardo Navarro-Contreras

**Affiliations:** 1 División de Gineco-Obstetricia, Hospital Central Dr. Ignacio Morones Prieto, Ave. Venustiano Carranza 2395, Zona Universitaria, 78290 San Luis Potosí, SLP Mexico; 2 Coordinación para la Innovación y Aplicación de la Ciencia y la Tecnología (CIACYT), Universidad Autónoma de San Luís Potosí, Álvaro Obregón 64, 78000 San Luis Potosí, SLP Mexico; 3 Department of Physics and Astronomy, University of Texas at San Antonio, San Antonio, TX 78249 USA; 4 Hospital General de Zona No.1, Instituto Mexicano del Seguro Social (IMSS), San Luis Potosí, SLP Mexico

**Keywords:** Sialic Acid, Surface-enhanced Raman, Ag Nanoparticles, Ovarian Cancer

## Abstract

**Background:**

To demonstrate the use of surface-enhanced Raman spectroscopy (SERS) to determine sialic acid (SA) levels in saliva using silver nanoparticles as substrates, in adnexal mass patients scheduled for surgical intervention to remove invasive masses, with the aim to compare SA levels in benign tumor vs ovarian cancer patients.

**Methods:**

Quantification of SA levels was accomplished by measuring their SERS and calibrating with analytical reagent SA. The mean SA concentration in saliva from 37 benign adnexal mass resulted smaller (5.1 mg/dL) than the mean concentration in 15 Ovarium cancer patients (23 mg/dL). The cancer condition was determined by biopsy of the removed adnexal mass. The CA-125 biomarker was also measured. The predictive potential of both biomarkers is discussed, together with the malignity risk index (MRI).

**Results:**

Our results showed a sensitivity/specificity of 80%/100% with a cutoff to distinguish between benign/cancer cases of SA 15.5 mg/dL, as established from a ROC analysis. Our results suggest that SA may be a more useful biomarker than CA-125 to detect ovarian cancer.

**Conclusions:**

Our results suggest that the SA levels measured from saliva may be as good predictors as the MRI index for the presence of ovarian cancer in sensitivity/negative predictive value and outperforms it in specificity/positive predictive value.

**Electronic supplementary material:**

The online version of this article (10.1186/s13048-018-0433-9) contains supplementary material, which is available to authorized users.

## Background

Ovarian cancer is the seventh most common cancer in women worldwide [1, 2]. In 2012 239,000 ovarian cancer cases were reported; which amounts to 4% of all new cases of cancer in women. Ovarian cancer produced approximately 152,000 deaths in 2012. It is the eighth most common cause of cancer death in women across the world [1, 2].

Ovarian cancer (OC), in general do not produce symptoms at early stages. Additionally, there is no early detection method applicable to the general women population, so the disease is generally advanced when it is diagnosed. Ovarian epithelial cancer is the histological type of major incidence, representing almost 90% of reported cases. In more than 70% of all cases, it is usually detected in the advanced clinical stages III and IV. The 5 year survival rate ranges from approximately 30 to 50% depending on the type of ovarian cancer, being the invasive epithelial ovarian cancer the most common, as well as that of the worst prognosis, in phase III-IV patients, which have a 5–20% five year survival rate [3, 4].

This high mortality rate prompts the need to have effective, highly sensitive diagnostics methods to examine women who may have evidence of ovarian cancer, or symptoms who may lead the medical specialist to consider further clinical examination of the involved patient, to discard the existence of ovarian cancer.

For the medical diagnosis, there is a limited set of analysis that may help to detect and diagnose ovarian cancer. Transvaginal ultrasound is the most useful imaging method for routine screening of ovarian cancer, given that this cancer is usually only palpable in advanced stages. The most important biomarker that has proven to be a useful adjunct for ovarian cancer is the protein CA-125, which has been found is produced by more than 90% of advanced epithelial ovarian cancers [3, 4]. Normal values range from 0 to 35 (U/mL). As a result, the CA-125 protein has become the most screened serum marker in laboratory tests for ovarian cancer. However, although CA-125 significantly rises 4 months previous to any clinical manifestation, in almost 50% of the early screenings of positive cases, it results in normal concentration levels [5]. This fact reduces the usefulness of CA-125 as a predictive test for this malignancy. Additionally, for young patients (premenopausal women) there is not any clearly defined cutoff point for this biomarker which can be associated with any malignity risk with certainty.

Considering these limitations, as well as the fact that ovarian cancer is of relative low prevalence, screening of the general sensitive female population for ovarian is not recommended [5], although less than 30% of patients with ovarian cancer are diagnosed at an early curable stage [6]. Instead, an annual gynecological exam with pelvic examination is recommended for preventive health care.

One major problem is that screening methods tend to find a large number of false positive cases. The combined use of transvaginal ultrasound and CA-125 tend to result in a higher sensitivity for ovarian cancer detection, but at an increased rate of false positive results [5, 6]. Unfortunately, malignancy can only be assessed with surgery. These could lead to unnecessary surgery in false cases, with the additional full inconveniency of the probability of complications due to surgery, as well as anxiety caused by false alarming results.

Another biomarker for ovarian cancer screening that has been studied and proposed is the Human Epididymis Protein 4 (HE4) [7]. HE4 is a tumor marker of ovarian cancer, with 80% sensitivity at a cutoff of 150 pmol/L [7]. However, it is not routinely used by the medical professionals in standard medical tests to screen for the presence of this disease.

Saliva has been proposed as an alternative medium to plasma in monitoring biomarkers that may be useful in diagnosis of several human diseases, as well as an element to monitor the etiology of some pathologies or the effects of drugs doses. The simplicity and non-invasiveness of its recollection, the existing positive correlation among multiple substances that are present in serum and saliva, are some of the advantages that this fluid offers as a diagnostic instrument for some human diseases [8, 9].

An important biomarker in saliva is sialic acid (SA), which is a systematic biomarker of systemic inflammation [10], it is a component bound to salivary glycolipids, and to glycoproteins, of which IgA (immunoglobuline A) may be one of the most important, as well as from some other immunologic proteins. SA occupies the interface between a host and pathogenic microorganisms.

Sialic acids are a family of nine-carbon acidic monosaccharides that occur at the end of oligosaccharide chains of mucins, glycoproteins, and glycolipids attached to the cell membranes of all vertebrate organisms [10]; SA emerging from these membranes is continuously shed into the surrounding human fluids. N-acetylneuraminic acid (Neu5AC) is the predominant form of SA and almost the only form found in human body fluids and tissues, among them saliva [10]. It has the chemical formula C_11_H_19_NO_9_. The molecule is composed of a pyranose ring consisting of five carbon atoms and one oxygen atom, to which one N-acetyl, one carboxyl group, and one glycerol ion are attached.

SA is normally detected and calibrated in its concentration in human fluids, by decanting it by an elaborated chemical process to isolate it from other proteins or non-related lipids which are constitutive of these fluids. Followed by comparison of its absorbance at some given wavelengths with standard calibration curves obtained from chemically pure reagents [11, 12]. Recently, we have shown that small amounts of SA in aqueous solution may be easily detected by the alternative method of surface enhanced Raman spectroscopy (SERS) [13], produced by citrate-reduced silver nanoparticles (cit-Ag-NP), with a simplified processing of the saliva sample. We have demonstrated the sensitivity of this method to distinguish between breast cancer patients and healthy control subjects [14]. This is possible as SA is well known to be over expressed in human fluids when there is an ongoing cancer development process. Determination of SA by SERS from cit-Ag-NP is a technological approach that requires fewer reagents, and simplified saliva sample processing. The latter constitutes an advantage that could be useful in clinical diagnosis since it is highly sensitive, fast, and low cost, moreover, the equipment may be portable and results can be obtained in real time.

SERS is a Raman spectroscopic technique that has shown enhancements of the inelastic scattering of the outgoing radiation ranging from 10^6^ to 10^12^ in amplification factors from molecules that have been adsorbed in metals [15]. Therefore, SERS has great potential as a molecularly specific analytic probe for highly sensitive detection of weak Raman signals of proteins or other biological analytes with low Raman scattering cross-section or at very low concentrations. The threshold of concentrations detectable may be as low as a single-molecule attached to a Ag-NP [15]. Furthermore, colloidal suspensions of metallic nanoparticles (NP), silver, gold or copper are the most common SERS substrates because they are of easy preparation and have long shelf-lifetime, and high Raman signal enhancement factors [16].

A primary aim of this study is to apply SERS to measure levels of SA in human saliva using colloidal suspension of cit-Ag-NP, and in particular to determine SA levels in saliva of patients to whom adnexal masses in ovary have been observed by ultrasound sonography, and who have been scheduled for surgical removal of the detected tumor.

### Hypothesis

The hypothesis is that there may exist a significant difference in SA levels which have been expressed in saliva, as in many other human fluids, between benign tumor cases and ovarian cancer affected patients.

Our main objective was to establish the usefulness of monitoring the SA levels by SERS from cit-Ag-NP, in suspected cases of ovarian cancer, thus adding the SA level tests as another adjunct probe to the diagnosis of ovarian cancer, together with CA-125 and ultrasound imaging. SERS has the advantages of its simplicity and may be extremely useful when it is necessary to monitor the progression of the disease as well as monitor the effectiveness of applied treatments. Our results corroborate that salivary SA concentrations are found to be significantly higher in ovarian cancer patients compared to that of benign adnexal mass patients.

## Methods

### The study population

Inclusion criteria: Patients attending the service of the Gynecology and Obstetrics at Central Hospital Dr. “Ignacio Morones Prieto” at San Luis Potosí, S.L.P. México, (HCDSLP) during the periods July–December 2017, which were diagnosed by ultrasound to have adnexal tumor masses and that were scheduled for surgical intervention to remove them, with no previous clinical treatment.

Exclusion criteria: any previous cancer diagnosis, second primary cancer, pregnancy, any systemic or circulatory diseases, or periodontal disease.

The study was approved by the HCDSLP Ethics Committee. Written informed consent was obtained from all participants.

### Cancer determination

Histological type determined by the Pathology laboratory at the Central Hospital “Dr. Ignacio Morones Prieto” from tumor biopsy.

### Data collection

Adnexal tumor patients completed a health questionnaire that included information about systemic health and oral diseases. Blind (regarding the existence of ovarian cancer) determination of the SA concentration (SAC) in saliva and CA-125 in serum were made.

### Saliva collection and processing

Before the saliva collection, each participant was required to perform a two-step oral cleansing. The first step consists of a vigorous teeth brushing and the second one involves two subsequent oral rinses with commercial alcohol-free mouthwash. The saliva samples were centrifuged at 6000 rpm (equivalent to 3580 G) for 15 min, and the resulting supernatants were used to determine the concentration of SA.

### SERS calibration of SA

The Raman measurements were performed on a Horiba Jobin Yvon XploRA ONE Raman spectrometer coupled to an Olympus BX41 optical microscope, using an excitation green laser 532 nm, with an average power of 20 mW at the sample. The laser beam was focused on the surface of the liquid sample with a 10X objective. The diameter of the laser spot was approximately 8–10 μm. The SERS spectra were collected from the 400 to 1800 cm^− 1^ spectral range. The intensity of three Raman shift lines, 1002, 1237 and 1391 cm^− 1^, after fluorescence subtraction, are used to compare with a calibration curve for the SERS obtained from SA Analytical Reagent grade. Next, to record the SERS spectra, 50 μl of a 2.5 × 10^− 3^ M citrate-reduced Ag-NP were placed in an aluminum container, 100 μl in capacity, mixed with 25 μl of the centrifuged saliva sample. An equal volume of a reference SA solution was using in the calibration process previous to any measurement session. Further details on the calibration process are provided in [14].

### Data statistical analysis

Continual variables are reported using the median ± interquartile range (IQR) because of the non-normal distribution of some of them, as discussed below. Categorical variables are reported as *n* and percentage. Non-parametric tests were performed to analyze the continual variables (Mann Whitney U test). The categorical variables were analyzed with the Fisher exact tests. The statistical analysis was carried out with the statistical program R 3.4.3 (R Core Team 2017), and RStudio Version 1.1.423. Receiver operating characteristics (ROC) curves were plotted to calculate the area under the curve and to establish the best point for sensitivity and specificity.

## Results and discussion

Fifty-two patients that were diagnosed with adnexal masses scheduled for surgical intervention, age ranging from 25 to 78 years old (average: 41.6 years ±17.3) participated in this study. The characteristics of the patients participating in the project are shown in Table 1. One may see that the histological analysis resulted in 37 patients with benign adnexal masses (mean age and S.D. 36.8 years ±15.3). In 15 patients (mean age and S.D. 51.2 years ±18.7), these masses were ovarian cancer.

**Table 1:** Patients that have been diagnosed with adnexal masses characteristics that were included in the study

Mann Whitney U test results	BAMP; *N* = 37		*Q1*	*Q3*	OCP; *N* = 15		*Q1*	*Q3*	*p* value
	Mean	Median			Mean	Median			
CA125 (U/mL)	80.6	34	21	54	1036.3	320	76.6	1662	0.0003
SAC (mg/dL)	5.1	3.2	2.3	7.3	23.0	27.4	18.6	28.5	0.0000
	Median				Median				*p* value
BMI (Kg/m^2^)	28		25	32	25		23	27	0.0745
Age Menarche	13.4		12	15	13		12	14	0.7491
	Mean		S.D.		Mean		S.D.		
Gestations	1		2		2		3		0.6524
Age	36.8		15.3		51.2		18.7		0.0148
Pos-menopausal	6 (16.2%)				8 (53.3%)				
Irregular menstruations	6 (16.2%)				2 (13.3%)				
MRI > 200 n (%)	4 (10.8%)				12 (80%)				< 0.0001

The *p* stands for the significance obtained for BAMP benign adnexal mass and OCP ovarian cancer affected patients, under the Mann Whitney U tests

A Shapiro test for normality in the distribution of the measured variables was performed. In the test, a significance level below *p <* 0.05 indicates that the variables are not normally distributed. Only body mass index and Menarche were normally distributed, the SA and CA-125 data require a non-parametric statistical analysis. Given the fact that most variables are non-parametric, we proceeded to evaluate the median plus/minus the first and third quartiles (*Q1, Q3*), which are summarized in Table 1 for the relevant parameters SAC and CA-125.

**Fig. 1:**
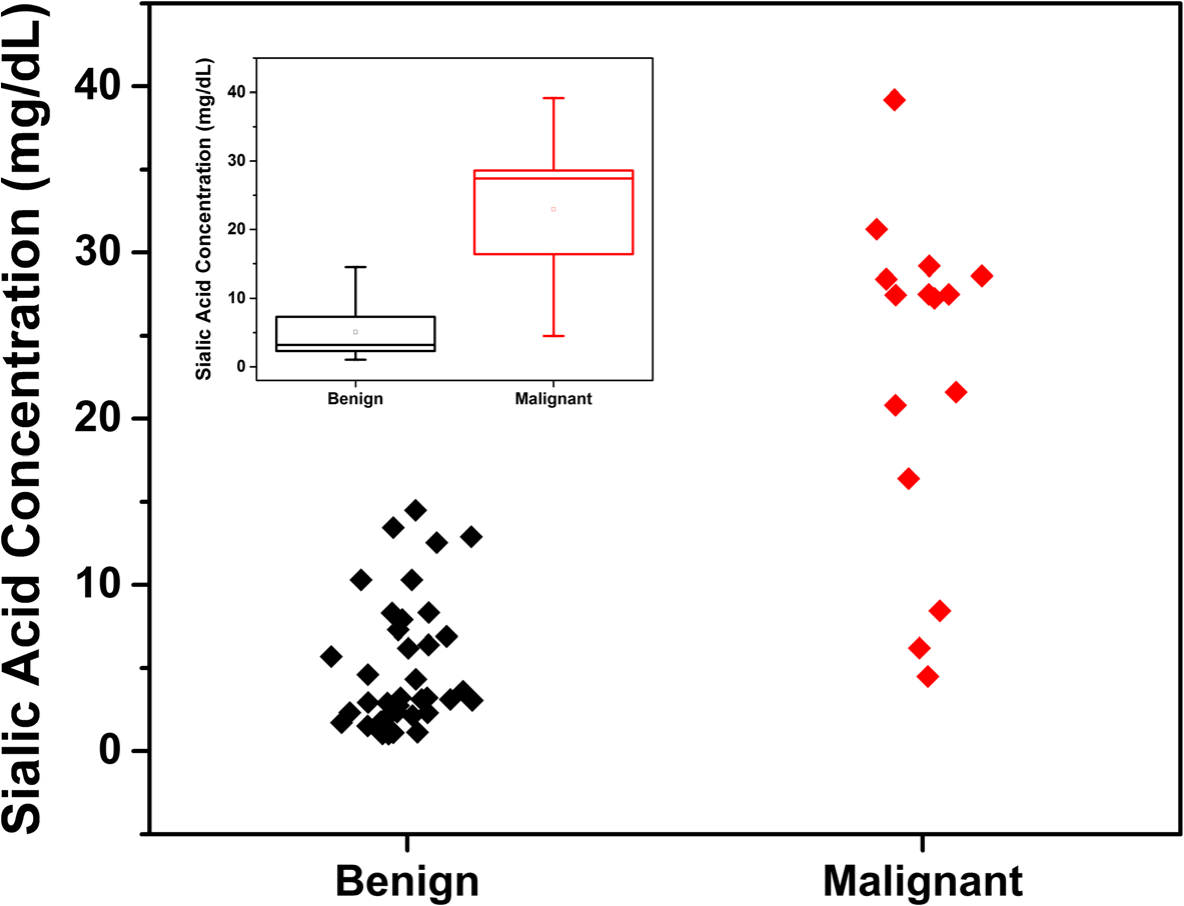
Cloud plot of sialic acid concentration of each group of patients. Red: ovarian cancer; black: benign adnexal mass. Insert: Box plot of sialic acid concentration, again red: ovarian cancer; black: benign adnexal mass

Figure 1 shows the sialic acid concentration (SAC) of each group of patients. Indicated in black, are the SAC of benign adnexal mass (BAMP) affected patients, and in red those in which histology has diagnosed ovarian cancer (OCP). The inset in Fig. 1 shows the boxplot of sialic acid concentration in both groups.

The difference between concentration means of benign adnexal mass patients and positive diagnosis ovarian cancer cases was assessed by a Mann Whitney U test, which has been also summarized in Table 1. A *P* ≤ 0.05 value was considered to be statistically significant. The application of the Mann Whitney U test supports the hypothesis that the expression of SA that is present in the saliva of benign and ovarian cancer patients is statistically different.

Table 1 shows that there are no expected statistical differences in the BMI, menarche and gestations variables between the two groups, BAMP and OCP. However, the same analysis indicates that there are significant statistical differences in both CA125 and SAC, between the two groups of patients.

**Fig. 2:**
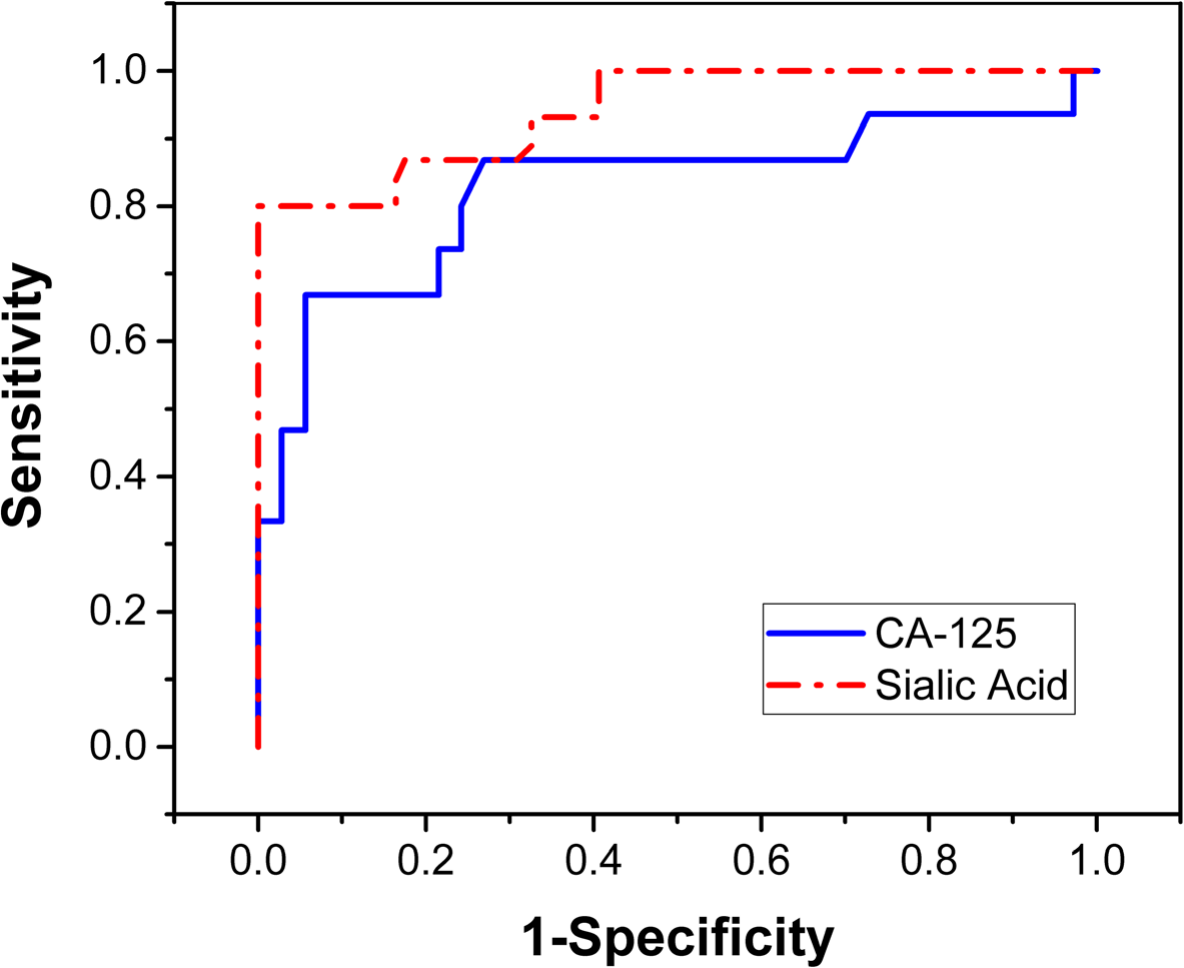
ROC curve analysis for SA (and CA-125), to optimize the threshold of acid sialic concentrations to distinguish between benign adnexal mass patients, and ovarian cancer patients

Figure 2 shows the result of a Receiver Operating Characteristic or ROC curve analysis for the sialic acid concentrations between the two group of patients. As always the axes are chosen vertical for Sensitivity (true positive rate), and horizontal 1-Specificity (false positive rate). This analysis performed with the R-Studio program results in that the best threshold to differentiate between BAMP and OCP corresponds to SAC > 15.5 mg/dL, rounded to one decimal digit. The area under the ROC curve is 94.05% for the SAC cutoff proposed. This SAC threshold value corresponds to a sensitivity of 80%, and specificity of 100%. The figure has also included the ROC analysis for the CA-125. This analysis results in a smaller area under the curve of 82.52%, as compared to the SAC. The resultant suggested cutoff from the ROC analysis for the CA-125 is 216.5 U/mL. This is a very high concentration value, corresponding to a high specificity value of 94.5%, but to a poor sensitivity because it fails to identify at least 8 out of the 15 positive cases (more than 50% of the cases).

**Fig. 3:**
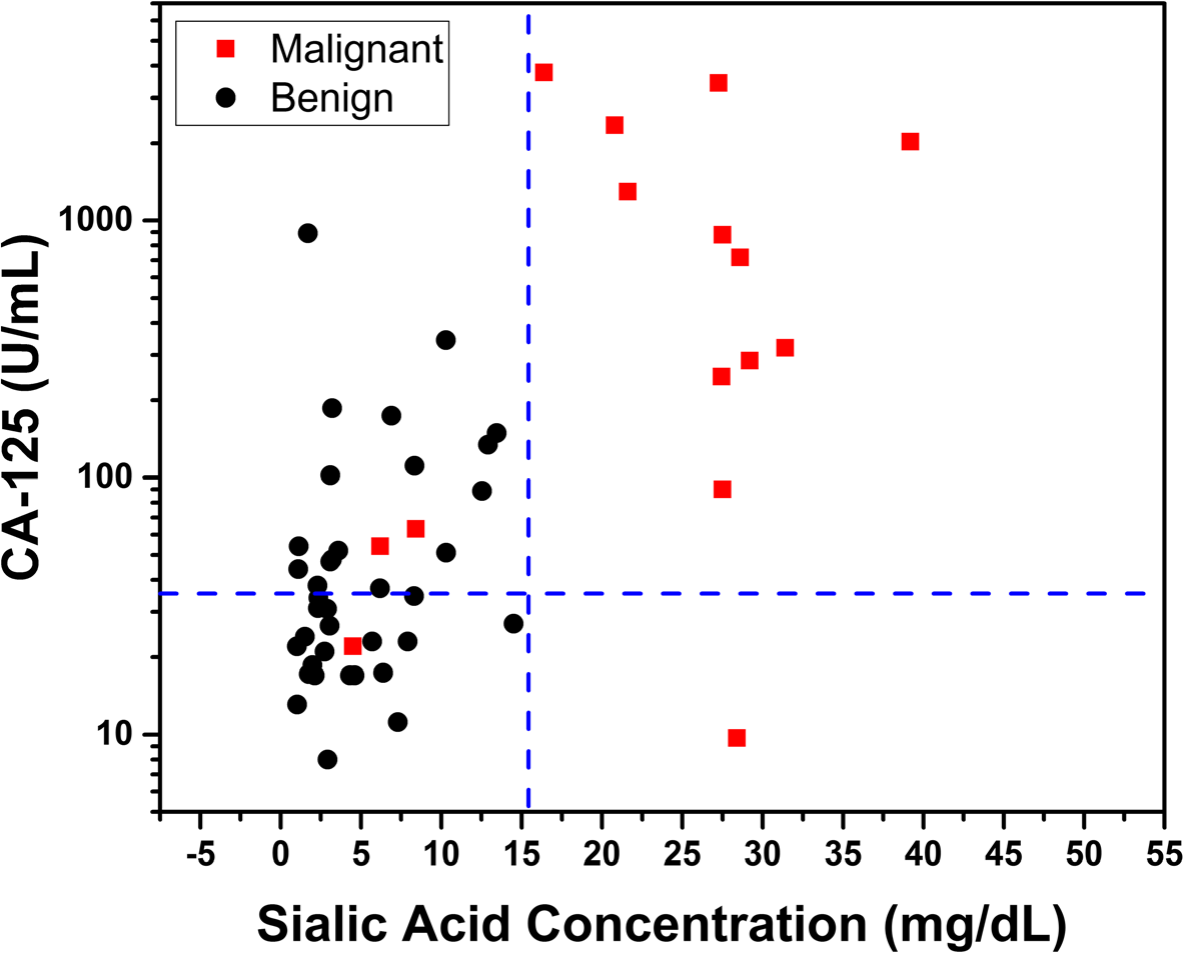
SA and CA-125 plot, to illustrate how the cutoff values of SA and CA-125 concentrations distinguish between benign adnexal mass patients (black), and ovarian cancer patients (red)

Figure 3 shows a CA-125 vs SA plot of the benign adnexal mass and ovarian patients, (black and red points, respectively). The Spearman correlation coefficient among CA-125 and SAC is 0.56, indicative of a modest correlation among the expression of these two biomarkers. The blue colored lines depict the standard cutoff concentrations for the CA-125 (horizontal dashed line, 35 U/mL) and the proposed SAC cutoff (vertical dashed line). The CA-125 concentrations are plotted on a logarithmic scale, in order to have a better visualization of the spreading in resultant patient concentrations that were determined for this biomarker. From the plot, it is evident that SA under the cutoff provided by the ROC analysis has a better performance than CA-125 to distinguish between benign and ovarian cases. But with respect to detecting true positives, CA-125 in this exercise missed only two positive cases, at the 35 U/ml cutoff, vs 3 for SA, but at the expense of resulting in too many false positives, 15 of them in this study (almost 40% of benign cases), against none for the SA, under the cutoff of 15.5 mg/dL.

**Fig. 4:**
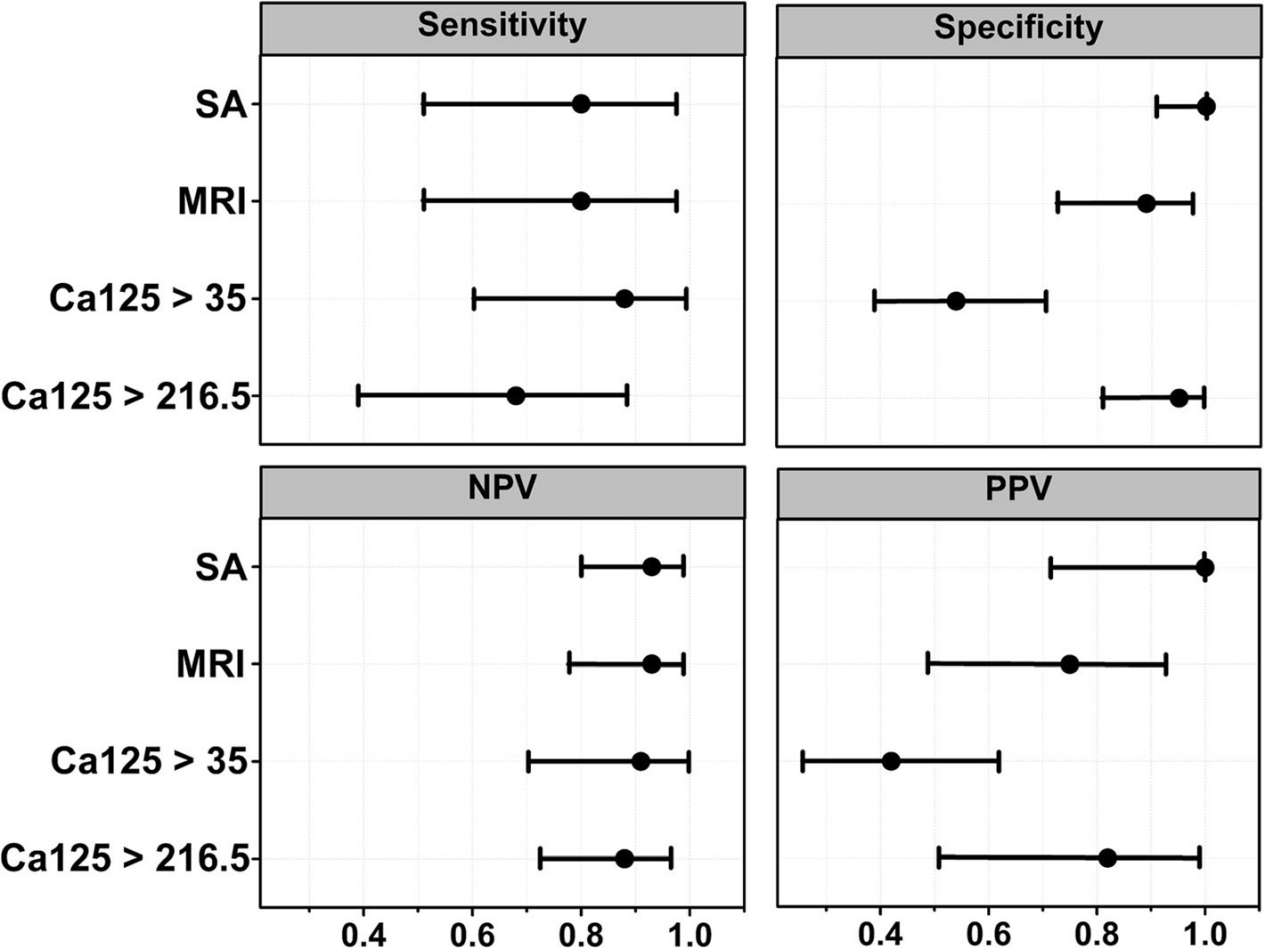
Sensitivity, specificity, positive predictive value (PPV) and negative predictive value (NPV) for each test. The Figure includes cutoff values and confidence intervals for each biomarker, as well as for the calculated MRI. Abbreviations: SA, sialic acid; MRI, Malignancy risk, index, CA125 > 35, CA-125 larger than 35 U/mL; CA125 > 216.5, CA-125 larger than 216.5 U/mL

Figure 4, presents a comparison between the sensitivities, specificities positive predictive values (PPV) and negative predictive values (NPV) for each test. The Figure includes the cutoff values and confidence intervals for each biomarker, as well as for the calculated MRI. The MRI was calculated following Jacobs et al. [17] *MRI = U × M × CA-125*. In this formula *M* is the menopausal status score, and *U* the evaluation of the ultrasound score [17]. The Figure shows how the SAC under the cutoff ROC value of 15.5 mg/dL performs as well as the calculated MRI in sensitivity and negative predictive values and may be even superior in specificity and positive predictive values. The Figure includes the performance of CA-125 under the cutoff values of 35 and the best result of the ROC analysis of our data of 216 U/mL.

## Discussion

The results indicate that with a cutoff or threshold value of 15.5 mg/dL of the SA concentration measured in saliva we have a 100% specificity (80% sensitivity). These results suggest that we could distinguish before surgery if an adnexal masses patient, as revealed through sonography, is going to have ovarian cancer with high probability, if the patient presents higher SA concentration levels than this cutoff value. Of course this analysis requires that patients are discarded of suffering any systemic or circulatory diseases, advanced periodontitis or from any other primary diagnosed cancer, to circumvent the intrinsic limitation that SA is a nonspecific marker of inflammation.

It is clear that although SA is not a specific marker for ovarian cancer, it should be considered in the clinical scenario of the adnexal mass diagnosed patient, and not in the population in general. In spite of the non-specificity for cancer, the values obtained in the tests suggest that they are sensitive and specific enough to differentiate an inflammatory process against a cancer process, when combined with the clinical evaluation by the medical professional.

It is important to note that in our study, the mean age of the benign adnexal mass group is 36.8 years old which is smaller than the mean age of the ovarian cancer group of age 51.2. It has been reported that the SA levels increases only by 4–5 and 7% in the age groups 50–59 and 60–69, compared to the 20–49 age group, in which the SA levels remain practically the same [18]. Hence, as the mean ages of the two groups fall below < 50 years, any age effect is not expected to affect in any significant way the SAC data of the patients in this study.

The search for malignant tumor markers has been a permanent concern for the professionals of gynecological oncology [18]. In particular for Ovarium cancer Schwartz et al. in 1987, calibrating from absorbance curves, they found that lipid-associated sialic acid (LSA) levels in the serum of patients without any clinical evidence of disease were statistically different, from those patients with evidence of the disease [19]. The medians of LSA that they reported are of 18.4 and 27.2 mg/dL, for those two groups of patients, respectively. Additionally, the Schwartz group [19] found that the simultaneous use of the LSA and CA-125 concentrations enhances significantly the sensitivity for Ovarium cancer detection, from 76% for CA-125 alone to 84% when using information of the two markers combined, with cut off values of LSA > 24 mg/dL and CA-125 > 60 U/mL. A similar improvement is also reported for the specificity of both tests combined. Petru et al. [20], studied also the use of CA-125 and LSA extracted from serum, to test for the increase in sensitivity for diagnosing Ovarium cancer. However, in this study with the determination of both markers no significantly improved sensitivity was reported, in contrast to the use of CA-125 alone. But Petru et al. [20] reports very high sensitivities values for CA-125 (90.2%) for diagnosing Ovarium cancer. These high values have not been replicated by other studies [21–23]. For instance, Li et al. [21], found that the combined determination of SA with hydroxyproline provides a better diagnosis value than either CA-125 or HE4. S. Ghosh [22] discusses in a recent work, how SA in serum may also be used as a clinical predictor for Ovarium cancer. Finally, we add that Thakkar et al. [23] found that protein-bound sialic acid (PBSA) provides the highest overall ability to discriminate between ovarian cancer patients and healthy subjects, in comparison with other glycoproteins in serum [23].

Our present work adds to those previous reports the value of SA as a predictor of possible Ovarium cancer presence in adnexial mass diagnosed patients when it is detected by SERS in saliva in larger concentrations than the cutoff value obtained in our ROC analysis of 15.5 mg/dL. An important problem that our results for SAC in saliva may help to alleviate is the large number of unnecessary surgeries performed during the approach to treat the adnexal lesion. Our study is not a screening test since it was performed in a population with adnexal pathology, however our test of the over expression of SAC in saliva shows a very high specificity. We state the expectation that his may help to significantly decrease the number of unnecesary invasive surgical procedures in the population of these patients.

The present study was by necessity limited to the small group of adnexal mass patients expecting for treatment at HCDSLP during a six month period, and that were scheduled for surgical intervention to remove them. The very promising results for the SERS test determination of SAC from saliva, in specificity and sensitivity for ovarian cancer, determined in this work, suggest that it is necessary to continue the study to include a larger group of patients to corroborate our present results.

A necessary commentary in regards to SERS as a characterization technique of chemical biomarkers is that still has not been readily adopted by the clinical institutions. The only limitation that we perceive is that it is sensitive to the selection of prepared nanoparticles (NP), i.e. metal and full surface coverage of functionalization molecule types required to keep them in suspension, which require a careful study to find the most appropriated to the biomarker in consideration. But once these are established, the NP’s are easily reproducible, with repetitive SERS properties.

## Conclusions

Our measurements of SA by SERS produced by citrate-reduced silver nanoparticles of ovarian cancer affected patients indicate that these levels are increased with respect to the median SA level in adnexal mass subjects, corroborating previous results in the literature, where SA has been measured by conventional absorbance methods, which require extensive chemical processing of the samples. The threshold to differentiate between benign adnexal mass and Ovarium cancer patients is found to be SAC > 15.5 mg/dL, from a ROC curve analysis. This SA level threshold or cutoff value corresponds to a sensitivity of 80%, and specificity of 100%. Our results suggest that the SA levels that are measured from saliva samples may be as good predictors as the MRI index for the presence of ovarian cancer in sensitivity and negative predictive value and may outperform it in specificity and positive predictive value.

The SERS method requires no chemical elaboration of the saliva samples, and could be useful in clinical diagnosis since it is highly sensitive, fast, inexpensive, the equipment may be portable and results can be obtained in real time. Thus, the SERS method to determine the SA level in saliva may be used as an adjunct test to diagnose the presence of ovarian cancer, distinguishing between benign adnexal mass cases and ovarian cancer, and to assess the efficacy of post-surgical treatment.

## Additional file

Additional file 1: Patients data participating in the sialic acid determination. (XLSX 18 kb)

## Abbreviations

(Q1, Q3): First and third quartiles
BAMP: Benign adnexal mass affected patients
BMI: Body mass index
CA-125: Cancer antigen 125
cit-Ag-NP: Citrate-reduced silver nanoparticles
HCDSLP: Central Hospital Dr. “Ignacio Morones Prieto” at San Luis Potosí
HE4: Human Epididymis Protein 4 (HE4)
IgA: Immunoglobuline A
IQR: Interquartile range
LSA: Lipid-associated sialic acid
MRI: Malignity risk index
Neu5AC: N-acetylneuraminic acid (sialic acid)
NP: Nanoparticles
NPV: Negative predictive values
OC: Ovarian cancer
OCP: Ovarian cancer patients OCP
PBSA: Protein-bound sialic acid
PPV: Positive predictive values
ROC: Receiver operating characteristics curves
S.D.: Standard deviation
SA: Sialic acid
SAC: Sialic acid concentration
SERS: Surface-enhanced Raman spectroscopy
*U*: Evaluation of the ultrasound score
